# The association of hepatic steatosis and fibrosis with heart failure and mortality

**DOI:** 10.1186/s12933-021-01374-8

**Published:** 2021-09-28

**Authors:** Jiyun Park, Gyuri Kim, Hasung Kim, Jungkuk Lee, You-Bin Lee, Sang-Man Jin, Kyu Yeon Hur, Jae Hyeon Kim

**Affiliations:** 1 Division of Endocrinology and Metabolism, Department of Medicine, Samsung Medical Center, Sungkyunkwan University School of Medicine, 81, Irwon-ro, Gangnam-gu, Seoul, 06351 Republic of Korea; 2 Data Science Team, Hanmi Pharm. Co., Ltd., Seoul, Korea; 3 Department of Clinical Research Design and Evaluation, Samsung Advanced Institute for Health Sciences and Technology, Seoul, Republic of Korea

**Keywords:** BARD score, Fatty liver index, Heart failure, Mortality, Nonalcoholic fatty liver disease

## Abstract

**Background:**

Nonalcoholic fatty liver disease (NAFLD) is a hepatic manifestation of metabolic disease and independently affects the development of cardiovascular (CV) disease. We investigated whether hepatic steatosis and/or fibrosis are associated with the development of incident heart failure (iHF), hospitalized HF (hHF), mortality, and CV death in both the general population and HF patients.

**Methods:**

We analyzed 778,739 individuals without HF and 7445 patients with pre-existing HF aged 40 to 80 years who underwent a national health check-up from January 2009 to December 2012. The presence of hepatic steatosis and advanced hepatic fibrosis was determined using cutoff values for fatty liver index (FLI) and BARD score. We evaluated the association of FLI or BARD score with the development of iHF, hHF, mortality and CV death using multivariable-adjusted Cox regression models.

**Results:**

A total of 28,524 (3.7%) individuals in the general population and 1422 (19.1%) pre-existing HF patients developed iHF and hHF respectively. In the multivariable-adjusted model, participants with an FLI ≥ 60 were at increased risk for iHF (hazard ratio [HR], 95% confidence interval [CI], 1.30, 1.24–1.36), hHF (HR 1.54, 95% CI 1.44–1.66), all-cause mortality (HR 1.62, 95% CI 1.54–1.70), and CV mortality (HR 1.41 95% CI 1.22–1.63) in the general population and hHF (HR 1.26, 95% CI 1.21–1.54) and all-cause mortality (HR 1.54 95% CI 1.24–1.92) in the HF patient group compared with an FLI < 20. Among participants with NAFLD, advanced liver fibrosis was associated with increased risk for iHF, hHF, and all-cause mortality in the general population and all-cause mortality and CV mortality in the HF patient group (all p < 0.05).

**Conclusion:**

Hepatic steatosis and/or advanced fibrosis as assessed by FLI and BARD score was significantly associated with the risk of HF and mortality.

**Supplementary Information:**

The online version contains supplementary material available at 10.1186/s12933-021-01374-8.

## Background

Nonalcoholic fatty liver disease (NAFLD) is a broad spectrum of liver disease that includes conditions ranging from steatosis to steatohepatitis, fibrosis, and cirrhosis in the absence of significant alcohol consumption or other known causes of liver disease [1, 2]. NAFLD is a well-known hepatic manifestation of metabolic disease and is also associated with carotid atherosclerosis and structural heart changes increasing the risk for cardiovascular (CV) disease (CVD) [3, 4]. Although controversy persists regarding the contribution of NAFLD to increased CVD in terms of whether it is a driving factor or cofactor, it is believed that patients with NAFLD experience more CVD and that the mechanism may be related to insulin resistance, oxidative stress, inflammation, endothelial dysfunction, gut microbiota and altered lipid metabolism [5–8]. Furthermore, the presence of advanced liver fibrosis coexisting with hepatic steatosis is correlated with greater risk of all-cause mortality [9, 10] and CV mortality [11].

Several studies reported that imaging- or biopsy-proven NAFLD/nonalcoholic steatohepatitis (NASH) was associated with subclinical changes to myocardial structure and/or function [12–14]. These structural or functional changes of the myocardium are thought to plan an important role in the progression of heart failure (HF); several cross-sectional studies revealed hepatic steatosis and fibrosis to be associated with clinically diagnosed HF [15, 16]. However, few longitudinal studies have evaluated the association between NAFLD and clinically diagnosed HF in the general population. Diagnosis of NAFLD including fibrosis staging relies on liver biopsy, which is not easy to conduct in a large population. Therefore, a non-invasive scoring system was proposed to stratify patients at high risk for hepatic steatosis and fibrosis. The fatty liver index (FLI), a surrogate marker of fatty liver, was validated in large population including Koreans [17, 18] and BARD score was developed to exclude advanced liver fibrosis in patients with NAFLD in the United States; its effectiveness was also previously validated among Asians [19, 20]. Several studies evaluated the association between FLI and cardiovascular disease [21, 22]. Higher liver fibrosis scores such as fibrosis-4 (FIB-4) score or nonalcoholic fatty liver disease fibrosis score (NFS) were associated with increased rates of liver disease and overall mortality [23] and adverse fatal or nonfatal CV events in patients with cardiometabolic disease [24]. However, few studies have investigated the association between FLI and clinically diagnosed HF both a general population and patients with pre-existing HF. In addition, the association of hepatic fibrosis defined using BARD score with HF outcomes and mortality has not been evaluated for those patients.

This study evaluated the association of hepatic steatosis using FLI and fibrosis using BARD score with the development of incident HF (iHF), hospitalization for HF (hHF), all-cause mortality, and CV mortality in a nationwide general population as well as a group of patients with pre-existing HF.

## Methods

### Data sources

We used the Korean National Health Insurance (NHIS) datasets of claims and preventive health check-ups in Korea from January 2009 to December 2012. The claims database includes primary and secondary diagnosis statements as defined by International Classification of Disease 10th revision (ICD-10) codes and detailed statements about prescription, procedures, hospital visits, and hospitalization [25]. The health check-up database contains questionnaires on lifestyle and behaviors as well anthropometric and laboratory measurements. Detailed methods regarding these measurements were described in previous research [26, 27]. We also used nationwide death certificate data from the Korean National Statistical Office. This study was approved by the institutional review board of Samsung Medical Center (approval no. SMC 2019-11-051), Seoul, Republic of Korea, who granted an exemption to the need for informed consent because all data provided by the NHIS to researchers were de-identified.

### Study population

**Fig. 1:**
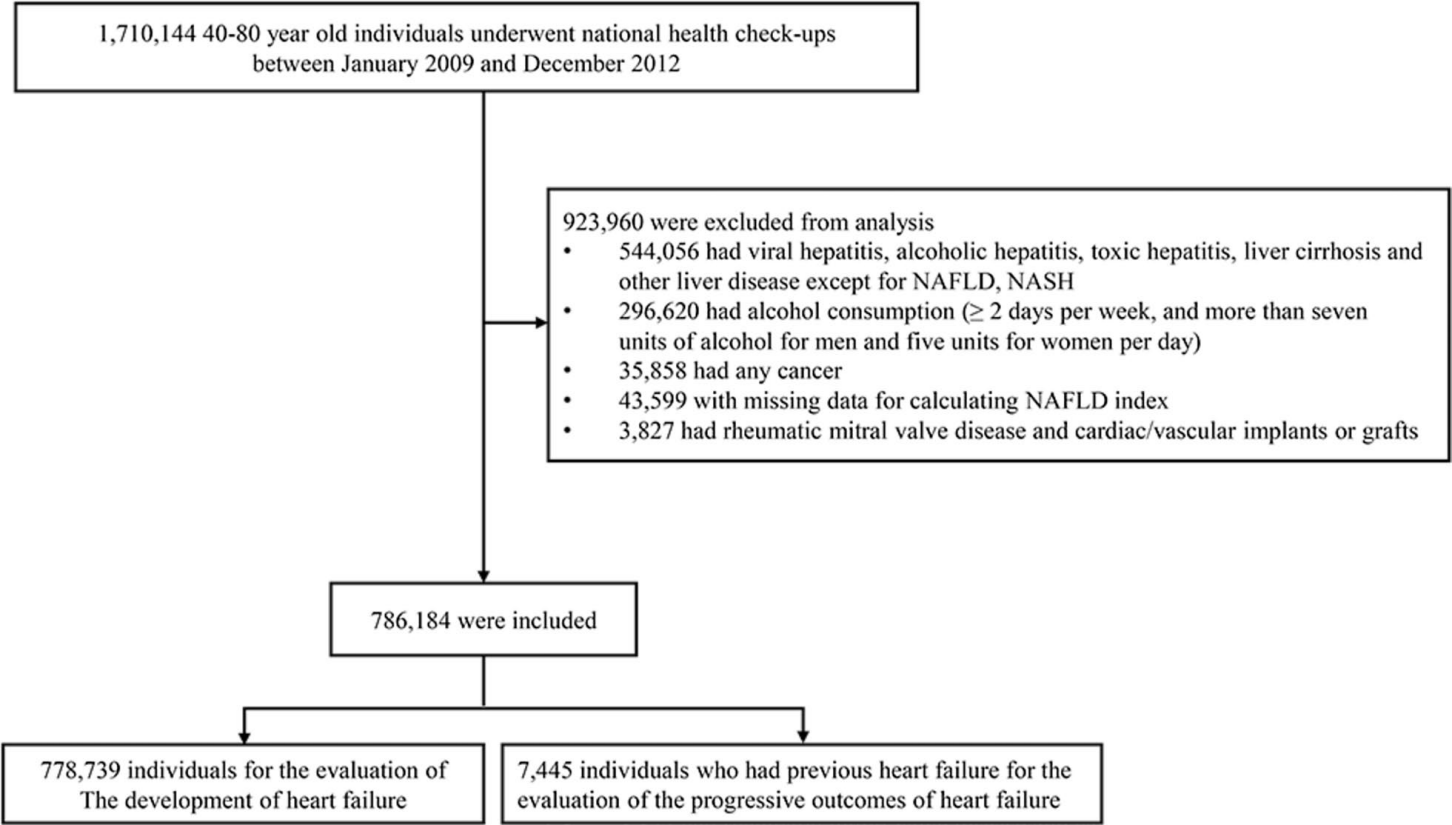
Flow diagram of the study population

Among all individuals aged 40 to 80 years who underwent regular health check-ups from January 2009 to December 2012, 10% were selected based on their age and sex (n = 1,710,144). For those who underwent more than two check-ups during this period, data from the first check-up were set as the baseline values. Among 1,710,144 subjects, we excluded 923,960 patients who had hepatitis and liver disease other than NAFLD, consumed alcohol at least 2 days per week and/or consumed more than seven units of alcohol for men or five units for women per day (daily unit × number of times per week ≥ 14 in men and ≥ 10 in women) [21], had any cancer, had rheumatic mitral valve disease or cardiac/vascular implants or grafts, or had missing data (Fig. 1). Among a total of 786,184 individuals, 778,739 subjects were analyzed to investigate the relationship between NAFLD and iHF. The relationship between NAFLD and progressive outcomes of pre-existing HF was examined in 7445 subjects with HF at baseline.

### Definitions of outcomes

The study endpoints were the development of iHF, hHF, all-cause mortality, and CV mortality. An iHF diagnosis was defined at the first hospital visit out of at least two or more outpatient hospital visits or first event of hHF [28]. Using claims data, we included both primary and secondary diagnoses of HF made during an outpatient hospital visit or hospitalization according to the ICD-10 disease code (I50). hHF was defined based on the first hospitalization with a primary diagnosis using ICD-10 code I50 [29]. CV mortality was defined as death caused by myocardial infarction (MI), HF, or hemorrhagic or ischemic stroke based on relevant ICD-10 codes (I21–I25, I50, I60, I61, I63, or I64) [30, 31].

### Measurements and definitions of variables

Information on current smoking, alcohol consumption, and regular exercise was collected from questionnaires. Regular exercise was determined as high-intensity physical activity (physical activity causing extreme shortness of breath) performed for at least 20 min at least three times per week or moderate-intensity physical activity (physical activity causing substantial shortness of breath) performed for at least 30 min at least five times per week. Income level was divided by quartile based on monthly income and the proportion of the lowest quartile was presented. Body mass index (BMI) was calculated as the participant’s weight in kilograms divided by their height in meters squared. Estimated glomerular filtration rate (eGFR) was calculated using the Chronic Kidney Disease Epidemiology Collaboration (CKD-EPI) equation. Fasting glucose and lipid measurements were obtained after an overnight fast. Metabolic syndrome was defined using the 2005 revision of the National Cholesterol Education Program (NCEP) criteria with Asian-specific cutoff values for abdominal obesity (i.e., waist circumference (WC) of 90 cm or greater in men or 80 cm or greater in women) [32]. Diabetes mellitus (DM) was defined as (i) at least one claim per year using ICD-10 codes E10 to E14 and at least one claim per year for the prescription of antidiabetic medication or (ii) by a fasting glucose level of at least 126 mg/dL. Hypertension was defined as (i) at least one claim per year using ICD-10 codes I10 or I11 and at least one claim per year for the prescription of antihypertensive agents or (ii) by a systolic/diastolic blood pressure of at least 140/90 mmHg. Dyslipidemia was defined as (i) at least one claim per year using ICD-10 code E78 and at least one claim per year for the prescription of a lipid-lowering agent or (ii) by a total cholesterol level of at least 240 mg/dL [26].

### Definition of hepatic steatosis and advanced hepatic fibrosis

NAFLD was defined using the previously validated fatty liver prediction model, FLI [17, 18]. FLI was calculated according to the following equation as (e^0.95 × ^$$^{{\log_{{\text{e}}} \left( {{\text{triglyceride}}} \right)}}$$ ^+^ ^0.139 × BMI + 0.718 × ^$$^{{\log_{{\text{e}}} \left( {{\text{gamma-glutamyl}}\;{\text{transferase}}} \right)}}$$^ + 0.053 × WC − 15.745^)/(1 + e^0.95 × ^$$^{{\log_{{\text{e}}} \left( {{\text{triglyceride}}} \right)}}$$^ + 0.139 × BMI + 0.718 × ^$$^{{\log_{{\text{e}}} \left( {{\text{gamma-glutamyl}}\;{\text{transferase}}} \right)}}$$^ + 0.053 × WC − 15.745^) × 100. The patients were classified into a low-risk group if their FLI value was 20 points or less; into an intermediate-risk group if their FLI value was 21 to 59 points; or a high-risk-group if their FLI value was 60 points or greater, which was defined as the NAFLD group [33]. Among participants with NAFLD (FLI ≥ 60 points), the presence of advanced liver fibrosis was defined based on the BARD score, which was calculated by assigning points for the presence of an aspartate aminotransferase (AST)/alanine aminotransferase (ALT) ratio of 0.8 or greater (two points), BMI of 28 kg/m^2^ or greater (one point), and DM (one point), where a total score of two to four points indicates advanced hepatic fibrosis [19].

### Statistical analysis

Statistical analyses were performed using the SAS software program (version 9.4; SAS Institute, Cary, NC, USA). Continuous variables are expressed as mean ± standard deviation values. Categorical data are presented as numbers with percentages. Comparisons of baseline characteristics according to FLI value were performed using a one-way analysis of variance (ANOVA) for continuous variables and chi-square test for categorical variables. Kaplan–Meier curves were used to show the cumulative incidence of iHF, hHF, all-cause mortality, and CV mortality and differences between groups were evaluated using the log-rank test. Cox regression analyses were used to evaluate hazard ratios (HRs) and 95% confidence intervals (CIs) for incidence rates of outcomes (i.e., iHF, hHF, all-cause mortality, and CV mortality). For multivariable-adjusted analyses using FLI, model 1 was crude; model 2 was adjusted for age, sex, and body weight; model 3 was further adjusted for alcohol drinking, smoking, regular exercise, and income status; and model 4 was further adjusted for hypertension, DM, dyslipidemia, and eGFR. For BARD score multivariable-adjusted analyses, the same models were adjusted except for DM in model 4, because it is included in the calculation of BARD score. For analysis of a small number of events, Firth’s logistic regression of rare events was performed as a standard approach [34]. The p-values for interaction were evaluated through a stratified analysis by age (< 65 years vs. ≥ 65 years), sex, the presence of DM, hypertension, and metabolic syndrome, BMI (< 25 kg/m^2^ vs. ≥ 25 kg/m^2^), eGFR (< 60 mL/min/1.73 m^2^ vs. ≥ 60 mL/min/1.73 m^2^), and triglyceride (TG) (< 100 mg/dL vs. ≥ 100 mg/dL) to confirm the potential interaction among such variables. A p-value of less than 0.05 was considered statistically significant.

## Results

### Baseline characteristics of the study population

The baseline characteristics of a total of 778,739 individuals without pre-existing HF according to their FLI cutoff value are summarized in Table 1. The number of individuals (n, %) in the FLI risk (low, intermediate, and high) groups were 432,445 (55.5%), 247,002 (35.2%), and 72,292 (9.3%), respectively. Variables such as BMI, waist circumference, fasting plasma glucose, gamma-glutamyl transferase (GGT), total cholesterol, TG, AST, ALT, and the proportion of patients with DM, hypertension, dyslipidemia, or metabolic syndrome increased as FLI score increased. Additional file 1: Table S1 summarizes baseline characteristics according to BARD score.

**Table 1:** Baseline characteristics of study population according to FLI score (n = 778739)

	FLI < 20	20 ≤ FLI < 60	FLI ≥ 60	p-value
n (%)	432,445 (55.5)	274,002 (35.2)	72,292 (9.3)	< 0.001
Age (years)	51.16 ± 9.87	54.44 ± 10.30	52.62 ± 10.06	< 0.001
Men (n [%])	112,354 (25.98)	145,704 (53.18)	49,559 (68.55)	< 0.001
Income level, lowest 25% (n [%])	113,174 (26.17)	64,123 (23.40)	16,994 (23.51)	< 0.001
Current smoker (n [%])	48,686 (11.26)	56,177 (20.50)	21,374 (29.57)	< 0.001
Regular exercise (n [%])	92,637 (21.42)	57,114 (20.84)	13,078 (18.09)	< 0.001
Body weight (kg)	56.12 ± 7.29	66.31 ± 8.28	76.28 ± 10.01	< 0.001
BMI (kg/m^2^)^a^	22.06 ± 2.17	25.22 ± 2.26	28.11 ± 3.07	
Waist circumference (cm)^a^	74.41 ± 6.19	84.56 ± 5.58	92.32 ± 6.75	
In men	77.50 ± 5.50	85.35 ± 5.14	92.05 ± 6.40	< 0.001
In women	73.32 ± 6.05	83.66 ± 5.91	92.91 ± 7.42	< 0.001
SBP (mmHg)	118.80 ± 14.80	126.29 ± 15.14	130.62 ± 15.57	< 0.001
DBP (mmHg)	73.82 ± 9.80	78.38 ± 9.91	81.61 ± 10.44	< 0.001
Fasting plasma glucose (mg/dL)	93.76 ± 18.22	101.02 ± 25.81	109.10 ± 34.03	0.046
AST (IU/L)	21.99 ± 11.78	25.17 ± 21.06	31.59 ± 24.34	< 0.001
ALT (IU/L)	18.02 ± 11.13	26.12 ± 20.10	39.58 ± 32.48	< 0.001
GGT (IU/L)^a^	18.20 ± 10.25	33.59 ± 27.06	68.86 ± 72.75	
Total cholesterol (mg/dL)	193.66 ± 37.22	206.51 ± 41.08	216.03 ± 44.62	< 0.001
Triglyceride (mg/dL)^a^	89.81 ± 40.15	156.59 ± 77.68	261.37 ± 189.55	
HDL-C (mg/dL)	59.00 ± 26.02	52.39 ± 30.36	50.78 ± 42.28	< 0.001
LDL-C (mg/dL)	118.03 ± 57.65	126.03 ± 60.70	123.01 ± 110.19	< 0.001
eGFR (mL/min/1.73 m^2^)	84.42 ± 20.64	75.72 ± 21.42	72.88 ± 21.84	
Comorbidities (n [%])				
Hypertension	81,620 (18.87)	103,291 (37.20)	35,356 (48.91)	< 0.001
Dyslipidemia	63,845 (14.76)	79,076 (28.86)	27,605 (38.19)	< 0.001
Diabetes mellitus^b^	19,635 (4.54)	31,756 (11.59)	14,077 (19.47)	
Metabolic syndrome^c^	53,214 (12.31)	127,973 (46.71)	56,321 (77.91)	< 0.001

Continuous variables are expressed as mean ± standard deviation. Categorical data are presented as frequencies and percentages

*AST* alanine aminotransferase, *ALT* aspartate aminotransferase, *BMI* body mass index, *DBP* diastolic blood pressure, *eGFR* estimated glomerular filtration rate, *FLI* fatty liver index, *GGT* gamma-glutamyl transferase, *HDL-C* high-density lipoprotein cholesterol, *LDL-C* low-density lipoprotein cholesterol, *SBP* systolic blood pressure

^a^p-values are not provided because these variables are included in the equation of FLI

^b^p-value is not provided because this variable is included in the equation of BARD score

^c^Metabolic syndrome was defined based on three or more of the following five risk factors: waist circumference ≥ 90 in men and ≥ 80 in women, triglyceride ≥ 150 mg/dL, HDL < 40 in men and < 50 in women, blood pressure ≥ 130/≥ 85, and fasting glucose ≥ 100 mg/dL

### Hepatic steatosis based on FLI and the incidence of HF and mortality in the general population

**Fig. 2:**
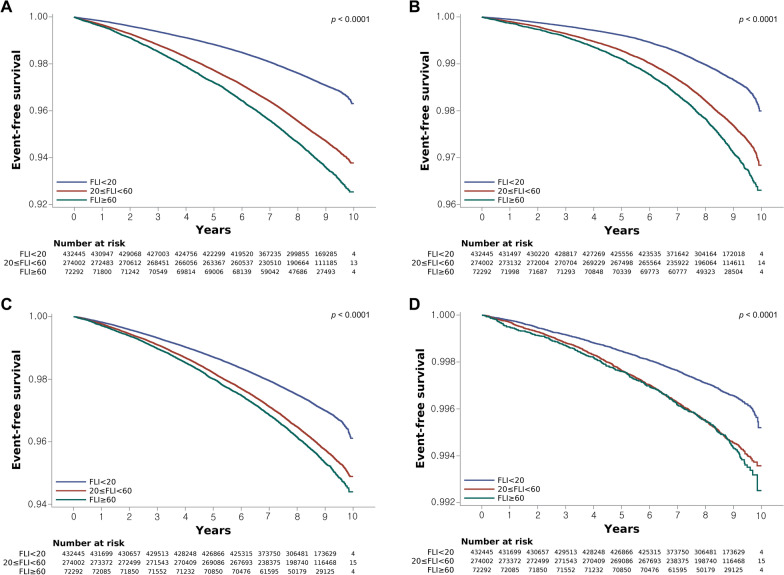
Kaplan–Meier estimates of incident heart failure, hospitalization for heart failure, all-cause mortality and CV mortality according to FLI. **A** Incident HF, **B** hospitalized HF, **C** all-cause mortality, and **D** CV mortality. *FLI* fatty liver index

During the median follow-up period of 8.5 years, 28,524 (3.7%) individuals developed iHF, 12,484 (1.6%) developed hHF, 25,667 (3.3%) experienced all-cause death, and 3074 (0.4%) subjects experienced CV death among a total of 778,739 subjects. Event-free survival for iHF, hHF, all-cause mortality, and CV mortality according to the FLI cutoff value using Kaplan–Meier curves is presented in Fig. 2. The cumulative incidence of HF, hHF, all-cause mortality, and CV mortality was significantly higher among participants with higher FLI cutoff values relative to those with FLI values of less than 20 points (p < 0.001, log-rank test) (Fig. 2). As indicated in Table 2, subjects with NAFLD (FLI ≥ 60 points) were significantly associated with an increased HR, 2.255 and 95% CI 2.176–2.337 (p < 0.001) for iHF in comparison with those without NAFLD (FLI < 20 points) (model 1, Table 2). After adjusting for age, sex, body weight, alcohol consumption, smoking, regular exercise, income status, hypertension, DM, dyslipidemia, and eGFR in model 4, a consistent result was also observed (adjusted HR [aHR] = 1.300, 95% CI = 1.240–1.362, p < 0.001), although the strength of association was partially attenuated (model 4, Table 2). In addition, the corresponding aHR (95% CI) values for hHF, all-cause mortality, and CV mortality were aHR 1.542 (1.438–1.655) 1.619 (1.540–1.702), and 1.413 (1.221–1.634), respectively (all p < 0.001). Analysis results on the risk of heart failure and mortality with FLI > 60 compared to 20 < FLI < 60 are presented in Additional file 1: Table S2. We further conducted subgroup analyses stratified by age, sex, hypertension, DM, BMI, eGFR, and triglycerides. The aHR and 95% CI values for iHF and all-cause death when comparing participants with NAFLD (FLI ≥ 60 points) to those without NAFLD (FLI < 20 points) according to these subgroups are presented in Additional file 1: Fig. S1. As compared with participants without NAFLD, those with NAFLD displayed a significant positive association for iHF and all-cause death in all subgroups analyzed.

**Table 2:** Hazard ratios and 95% confidence intervals for incident heart failure, hospitalization for heart failure, all-cause mortality, and cardiovascular mortality according to FLI score in the general population

	Model 1				Model 2			Model 3			Model 4		
	Events (n)	HR	95% CI	p value	HR	95% CI	p value	HR	95% CI	p value	HR	95% CI	p value
iHF													
FLI < 20	11,247	Ref.			Ref.			Ref.			Ref.		
20 ≤ FLI < 60	13,146	1.850	1.804–1.897	< 0.001	1.303	1.266–1.342	< 0.001	1.289	1.252–1.328	< 0.001	1.123	1.090–1.157	< 0.001
FLI ≥ 60	4131	2.255	2.176–2.337	< 0.001	1.715	1.683–1.795	< 0.001	1.670	1.595–1.749	< 0.001	1.300	1.240–1.362	< 0.001
hHF													
FLI < 20	5060	Ref.			Ref.			Ref.			Ref.		
20 ≤ FLI < 60	5630	1.738	1.673–1.805	< 0.001	1.334	1.276–1.394	< 0.001	1.305	1.249–1.364	< 0.001	1.160	1.109–1.213	< 0.001
FLI ≥ 60	1794	2.156	2.043–2.275	< 0.001	2.041	1.906–2.186	< 0.001	1.936	1.806–2.075	< 0.001	1.542	1.438–1.655	< 0.001
All-cause mortality													
FLI < 20	11,857	Ref.			Ref.			Ref.			Ref.		
20 ≤ FLI < 60	10,752	1.418	1.381–1.455	< 0.001	1.247	1.210–1.286	< 0.001	1.211	1.178–1.249	< 0.001	1.133	1.908–1.168	< 0.001
FLI ≥ 60	3058	1.557	1.496–1.620	< 0.001	1.992	1.897–2.091	< 0.001	1.858	1.768–1.952	< 0.001	1.619	1.540–1.702	< 0.001
CV mortality													
FLI < 20	1359	Ref.			Ref.			Ref.			Ref.		
20 ≤ FLI < 60	1351	1.554	1.442–1.676	< 0.001	1.326	1.214–1.447	< 0.001	1.287	1.178–1.405	< 0.001	1.105	1.011–1.207	0.028
FLI ≥ 60	364	1.616	1.440–1.814	< 0.001	2.015	1.748–2.323	< 0.001	1.871	1.620–2.160	< 0.001	1.413	1.221–1.634	< 0.001

*CI* confidence interval, *CV* cardiovascular, *HR* hazard ratio, *iHF* incident heart failure, *hHF* incident hospitalized heart failure

Model 1: crude

Model 2: age, sex and body weight

Model 3: model 2 + alcohol drinking, smoking, regular exercise, and income status

Model 4: model 3 + hypertension, diabetes mellitus, dyslipidemia, and estimated glomerular filtration rate

### Advanced hepatic fibrosis based on BARD score and the incidence of HF and mortality in patients with NAFLD

**Fig. 3:**
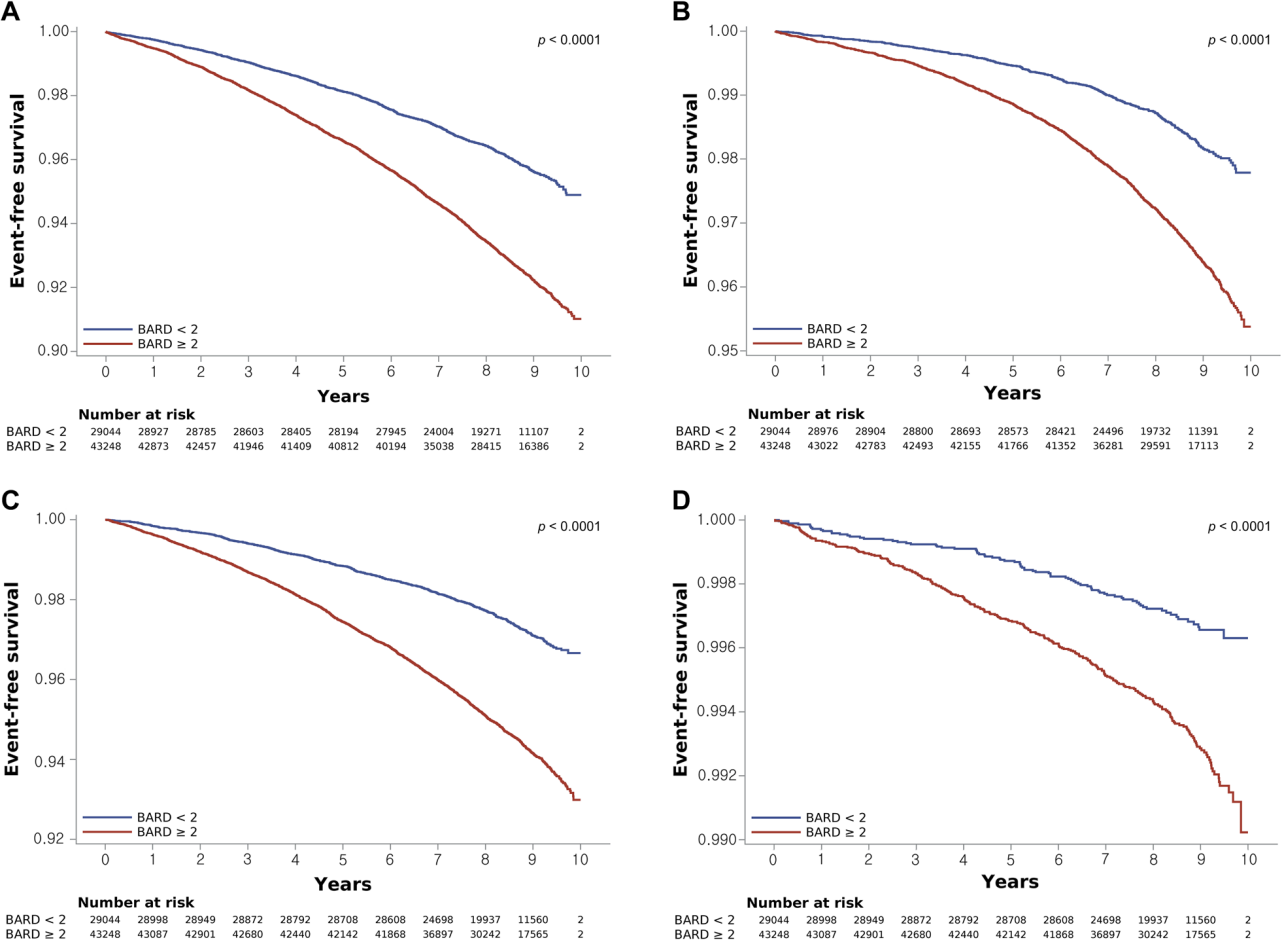
Kaplan–Meier estimates of incident heart failure, hospitalization for heart failure, all-cause mortality and CV mortality according to BARD score. **A** Incident HF, **B** hospitalized HF, **C** all-cause mortality, and **D** CV mortality

Among 72,292 patients with FLI values of 60 points or greater, 4131 (5.7%) had iHF, 1797 (2.5%) had hHF, 3058 (4.2%) experienced all-cause death, and 364 (0.5%) experienced CV death. Figure 3 shows the event-free survival for iHF, hHF, all-cause mortality, and CV mortality according to the presence of advanced hepatic fibrosis (BARD score ≥ 2 points). The cumulative incidence of HF, hHF, all-cause mortality, and CV mortality was significantly higher among participants with advanced hepatic fibrosis (p < 0.001, log-rank test) (Fig. 3). Table 3 shows that individuals with advanced hepatic fibrosis demonstrated significantly higher HR (95% CI) values for iHF (1.820, 1.698–1.950), hHF (2.083, 1.868–2.323), all-cause mortality (2.104, 1.935–2.288), and CV mortality (2.140, 1.677–2.731) relative to those without advanced hepatic fibrosis (all p < 0.001) (model 1, Table 3). The aHRs for these outcomes remained statistically significant in further multivariable models, except for CV mortality (model 4, Table 3).

**Table 3:** Multivariable Cox regression analyses showing the association of advanced liver fibrosis (defined by BARD ≥ 2) with the risk of incident heart failure, hospitalization for heart failure, all-cause mortality and cardiovascular mortality in patients with NAFLD (defined by FLI ≥ 60)

Advanced liver fibrosis	Model 1				Model 2			Model 3			Model 4		
	Events (n)	HR	95% CI	p value	HR	95% CI	p value	HR	95% CI	p value	HR	95% CI	p value
iHF	3039	1.820	1.698–1.950	< 0.001	1.124	1.045–1.209	0.002	1.136	1.056–1.222	< 0.001	1.116	1.037–1.201	0.003
hHF	1370	2.083	1.868–2.323	< 0.001	1.199	1.069–1.344	0.002	1.219	1.087–1.368	< 0.001	1.201	1.070–1.347	0.002
All-cause mortality	2342	2.104	1.935–2.288	< 0.001	1.235	1.132–1.348	< 0.001	1.248	1.143–1.362	< 0.001	1.240	1.135–1.353	< 0.001
CV mortality	280	2.140	1.677–2.731	< 0.001	1.217	0.943–1.571	0.132	1.262	0.977–1.630	0.075	1.237	0.957–1.598	0.104

*CI* confidence interval, *CV mortality* cardiovascular mortality, *HR* hazard ratio, *iHF* incident heart failure, *hHF* incident hospitalized heart failure

Model 1: crude

Model 2: age, sex and body weight

Model 3: model 2 + alcohol drinking, smoking, regular exercise, and income status

Model 4: model 3 + hypertension, dyslipidemia, and estimated glomerular filtration rate

### Hepatic steatosis based on FLI and the incidence of hHF and mortality in patients with established HF

Among 7445 patients with established HF, 1422 (19.1%) of the patients developed hHF, 1278 (17.1%) patients experienced all-cause mortality and 241 (3.2%) patients experienced CV mortality. Those with NAFLD were significantly more likely to experience hHF and all-cause mortality events compared with those without NAFLD in multivariable-adjusted analyses (hHF, aHR = 1.259, 95% CI = 1.027–1.543, p = 0.027; all-cause mortality, aHR = 1.541, 95% CI = 1.236–1.922, p < 0.001) (model 4, Table 4). However, the association between NAFLD and CV mortality was not statistically significant in the crude and multivariable-adjusted models (Table 4).

**Table 4:** Hazard ratios and 95% confidence intervals for hospitalization for heart failure, all-cause mortality and cardiovascular mortality in patients with previous heart failure according to FLI score

	Model 1				Model 2		Model 3				Model 4		
	Events (n)	HR	95% CI	p value	HR	95% CI	p value	HR	95% CI	p value	HR	95% CI	p value
hHF													
FLI < 20	495	Ref.			Ref.			Ref.			Ref.		
20 ≤ FLI < 60	677	1.059	0.943–1.189	0.334	1.123	0.982–1.283	0.090	1.113	0.973–1.272	0.118	1.040	0.909–1.190	0.567
FLI ≥ 60	250	1.141	0.980–1.328	0.090	1.469	1.203–1.793	< 0.001	1.428	1.168–1.746	< 0.001	1.259	1.027–1.543	0.027
All-cause mortality													
FLI < 20	525	Ref.			Ref.			Ref.			Ref.		
20 ≤ FLI < 60	563	0.823	0.730–0.926	0.001	1.151	1.001–1.325	0.049	1.141	0.991–1.313	0.067	1.054	0.915–1.215	0.466
FLI ≥ 60	190	0.803	0.680–0-948	0.010	1.836	1.480–2.277	< 0.001	1.792	1.441–2.228	< 0.001	1.541	1.236–1.922	< 0.001
CV mortality													
FLI < 20	93	Ref.			Ref.			Ref.			Ref.		
20 ≤ FLI < 60	114	0.942	0.717–1.239	0.671	1.355	0.982–1.870	0.064	1.338	0.968–1.850	0.077	1.210	0.874–1.675	0.250
FLI ≥ 60	34	0.812	0.549–1.203	0.300	1.937	1.170–3.206	0.010	1.850	1.112–3.077	0.018	1.535	0.918–2.568	0.102

*CI* confidence interval, *CV mortality* cardiovascular mortality, *HR* hazard ratio, *hHF* hospitalized heart failure

Model 1: crude

Model 2: age, sex and body weight

Model 3: model 2 + alcohol drinking, smoking, regular exercise, and income status

Model 4: model 3 + hypertension, diabetes mellitus, dyslipidemia, and estimated glomerular filtration rate

### Advanced hepatic fibrosis based on BARD score and the incidence of hHF and mortality in patients with established HF and NAFLD

Among 1202 patients with established HF and NAFLD (FLI ≥ 60 points), 250 (20.8%) patients developed hHF, 213 (17.7%) patients experienced all-cause death, and 33 (2.7%) patients experienced CV death. As shown in Table 5, as compared with participants without advanced hepatic fibrosis, those with advanced hepatic fibrosis were significantly more likely to experience all-cause death (aHR = 1.597, 95% CI = 1.001–2.548, p = 0.049) and CV mortality (aHR = 5.454, 95% CI = 1.047–28.400, p = 0.044), even after adjusting for multiple covariates, but hHF was not associated with advanced hepatic fibrosis (model 4, Table 5).

**Table 5:** Multivariable Cox regression analyses showing the association of advanced liver fibrosis (defined by BARD ≥ 2) with the risk of hospitalization for heart failure, all-cause mortality and cardiovascular mortality in patients with both NAFLD (defined by FLI ≥ 60) and previous heart failure

Advanced liver fibrosis	Model 1				Model 2			Model 3			Model 4		
	Events	HR	95% CI	p value	HR	95% CI	p value	HR	95% CI	p value	HR	95% CI	p value
hHF	213	1.456	1.027–2.065	0.035	1.162	0.811–1.665	0.413	1.127	0.786–1.616	0.517	1.089	0.758–1.565	0.644
All-cause mortality	169	1.994	1.267–3.138	0.003	1.615	1.017–2.566	0.042	1.656	1.038–2.639	0.034	1.597	1.001–2.548	0.049
CV mortality^a^	33	5.525	1.051–29.053	0.044	5.476	1.031–29.088	0.046	5.787	1.097–30.538	0.039	5.454	1.047–28.400	0.044

*CI* confidence interval, *CV mortality* cardiovascular mortality, *HR* hazard ratio, *hHF* hospitalized heart failure

Model 1: crude

Model 2: age, sex and body weight

Model 3: model 2 + alcohol drinking, smoking, regular exercise, and income status

Model 4: model 3 + hypertension, dyslipidemia, and estimated glomerular filtration rate

^a^Due to the small number of CV mortality events, Firth’s logistic regression was performed

## Discussion

In this nationwide, large population-based cohort study with a median follow-up period of 8 years, we evaluated the association of hepatic steatosis and/or advanced hepatic fibrosis using FLI and BARD score with iHF, hHF, all-cause mortality, and CV mortality among participants without HF as well as those with pre-existing HF. We found that NAFLD assessed by FLI was a significant risk factor for iHF, hHF, all-cause mortality, and CV mortality in those without established HF and an independent risk factor for all-cause mortality and CV mortality among patients with established HF. Moreover, we also demonstrated that advanced hepatic fibrosis assessed by BARD score was significantly associated with increased risk for iHF, hHF, and all-cause mortality among individuals without HF and was positively correlated with increased risk for all-cause mortality and CV mortality among patients with pre-existing HF. These results remained significant even after adjusting for other covariates such as body weight, hypertension, DM, dyslipidemia consist of metabolic syndrome. To the best of our knowledge, this study is the first to investigate the association between FLI and BARD score and HF outcomes in the general population and patients with pre-existing HF, separately, in a large study.

Recently, one study that analyzed the FLI values of healthy adults divided into four groups according to quartile reported that higher FLI values could predict the development of iHF [28]. Our study showed that classification of patients into three groups according to FLI cutoff values, which are widely validated to rule-out and rule-in NAFLD, revealed associations not only with iHF but also with hHF, all-cause mortality, and CV mortality in the general population. In this manner, we revealed a gradual association between higher FLI values and greater incidence of HF outcomes, all-cause mortality, and CV mortality, and also provided an exact aHR for HF outcomes when comparing the presence and absence of NAFLD. The CV mortality results in our study are consistent with those of a previous study evaluating the association between FLI and myocardial infarction, stroke and CV death in Koreans without previously established myocardial infarction or stroke [21]. In addition to CV mortality, NAFLD defined by FLI was also associated with hHF in patients with and without previous HF in our study. Prior studies mostly reported the incidence of iHF or hHF in participants without pre-existing HF, but our study further evaluated HF outcomes, including hHF, all-cause mortality, and CV mortality, in patients with established HF to suggest the clinical prognosis of HF. Considering that hHF is an important cause of mortality and re-hospitalization associated with major public health and economic burdens, it is important to identify and manage patients at high risk for HF. Furthermore, the presence of NAFLD needs to be carefully monitored in concert with the development of HF in the general population and considered as one of the poor risk factors for hHF and mortality in patients with HF.

NAFLD is usually accompanied by metabolic syndrome components; metabolic syndrome and type 2 diabetes are frequently associated with NAFLD. Due to shared pathophysiological aspects of insulin resistance, there is a close connection between NAFLD and other metabolic disease [35]. Recently, NAFLD has been shown to be an independent early predictor and determinant for development of metabolic syndrome, hypertension, and diabetes [1, 36–38]. Because some components of FLI, such as TG, BMI, and WC, are also included in the components indicating metabolic syndrome, there could be an association between FLI and metabolic syndrome. Patients with higher FLI tended to have more metabolic alterations compared to those with lower FLI at baseline in our study. To account for the effect of these metabolic components on our study outcomes, we adjusted for traditional CV risk factors such as body weight, hypertension, dyslipidemia and diabetes, and the independent significant association was not attenuated. We also observed that the association of FLI with iHF and mortality remained significant in groups with and without metabolic syndrome.

There was a significant association of FLI with iHF and mortality in both groups with diabetes and without diabetes. Individuals without diabetes had higher HRs of 1.33 and 1.81 than those with diabetes, who had HRs of 1.16 and 1.58 for iHF and mortality, respectively. Similar patterns were observed in subgroup analysis of metabolic syndrome. High FLI itself seemed to have a stronger effect on HF outcomes in people without diabetes or metabolic syndrome compared to those with diabetes or metabolic syndrome. Further studies regarding the magnitude of independent risk of metabolic alterations in HF are needed in the future.

Our study results showed that advanced hepatic fibrosis, defined by a BARD score of at least two points, was associated with iHF, hHF, and all-cause mortality among subjects with NAFLD. Although the association between advanced hepatic fibrosis and CV mortality was not statistically significant, a tendency toward increasing HR was observed. In the patients with established HF and NAFLD, both all-cause mortality and CV mortality were positively associated with advanced hepatic fibrosis. These findings are meaningful in that, in addition to the presence of NAFLD, advanced hepatic fibrosis is a strong predictor for HF outcomes and could be used as a screening tool to predict not only the development of HF and mortality in the general population but also all-cause and CV mortality in patients with established HF.

The precise mechanism linking hepatic steatosis and fibrosis with HF is poorly understood. Several hypotheses explaining the association suggest insulin resistance, subclinical inflammation, and dyslipidemia, as we previously mentioned [2, 5]. Advanced hepatic fibrosis is also associated with oxidative stress, which mediates inflammatory cytokines such as tumor necrosis factor-α and interleukin-6. These inflammatory processes can trigger functional and structural cardiac alternations, which may contribute to symptoms and long-term outcomes [39–41]. Measurable clinical findings of structural outcomes, such as diastolic dysfunction, left ventricular mass index, and valve calcification, have also been associated with hepatic steatosis and fibrosis, supporting the hypothesis mentioned above [42].

## Strengths and limitations

The strength of this study is that it is a large population-based longitudinal investigation and the first to evaluate the association between hepatic steatosis and/or fibrosis and HF outcomes using affordable and readily available biomarker-based models. We also studied not only a population without HF at baseline but also patients with pre-existing HF to assess both the development of HF and the progression of HF outcomes in the relationship with NAFLD. However, this study has several limitations. First, we used NHIS claims datasets, which include claims data reported based on diagnostic codes for diseases and outcomes, so misdiagnoses could be included. In addition, we could not evaluate heart failure phenotype due to the lack echocardiography or N-terminal fragment of the prohormone brain-type natriuretic peptide (NT-proBNP) data. Also, we could not assess symptoms or signs of HF using the NHIS claims dataset and HF diagnosis was defined based on diagnostic codes. Therefore, we set the definition of iHF and hHF using stricter criteria. Second, this was a retrospective analysis; therefore, causal relationships and unknown factors influencing the results were not evaluated. Third, the diagnosis of hepatic steatosis and fibrosis was based only on FLI and BARD score, not on imaging or biopsy. BARD score has a relatively low positive predictive value but the area under the receiver operating characteristics (AUROC) for prediction of advanced liver fibrosis exceeds 0.8 [19]. Because the Korean NHIS database did not include information about platelet count or albumin level, other liver fibrosis prediction scores such as FIB-4, NAFLD fibrosis score or Hepamet fibrosis score were not assessed. As liver enzymes could be elevated in patients with pre-existing HF, this could affect the relationship between liver fibrosis and HF outcomes. However, the BARD score uses the AST/ALT ratio instead of aminotransferase levels, BMI and DM. Previously, a high AST/ALT ratio was also found to be associated with the severity of HF, showing high NT-proBNP and low ejection fraction in relation to other liver fibrosis prediction models [43]. Fourth, although we adjusted for confounding factors in our results, unmeasured confounding factors might have been ignored. Finally, this study analyzed the Korean population, so it is unclear whether the results of this study could be generalized to other ethnic groups.

## Conclusion

NAFLD as defined by FLI value was associated with increased risk for iHF, hHF, all-cause mortality, and CV mortality in a general Korean population and was also associated with increased risk for hHF and all-cause mortality in patients with established HF. Furthermore, advanced hepatic fibrosis defined by BARD score was associated with increased risk for iHF, hHF, and all-cause mortality in patients with NAFLD and with increased risk for all-cause mortality and CV mortality in patients with NAFLD and established HF. HF is a growing public health concern due to associated mortality and healthcare expenditures. In addition, the prevalence of hospital readmission for HF continues to rise. Due to the lack of optimal pharmacologic options for HF, it is important to prevent development and slow progression of HF by finding novel risk factors that could be modifiable. This study suggests that hepatic steatosis and/or fibrosis is an independent risk factor of HF, and that its management may help prevent HF or improve HF outcomes.

## Supplementary Information

**Additional file 1: Table S1.** Baseline characteristics of study population according to BARD score in patients with NAFLD (defined by FLI ≥ 60) (n = 72,292). **Table S2: **Hazard ratios and 95% confidence intervals for the incident heart failure, hospitalization for heart failure, all-cause mortality and cardiovascular mortality for FLI ≥ 60 compared with 20 ≤ FLI < 60. **Figure S1.** Subgroup analysis for incident heart failure in patients with NAFLD (FLI ≥ 60) compared to those without NAFLD (FLI < 20). **Figure S2.** Subgroup analysis for all-cause death in patients with NAFLD (FLI ≥ 60) compared to those without NAFLD (FLI < 20).

## Data Availability

The data that support the findings of this study are available form Korean National Health Insurance Service (KNHIS), but restrictions apply to their availability. However, data are available from the authors upon reasonable request and with permission from the KNHIS.

## Abbreviations

aHR: Adjusted HR
BMI: Body mass index
CI: Confidence interval
CV: Cardiovascular
DM: Diabetes mellitus
FLI: Fatty liver index
HF: Heart failure
hHF: Hospitalized HF
HR: Hazard ratio
iHF: Incident HF
NAFLD: Nonalcoholic fatty liver disease
