# Supplementary material for: The association of hepatic steatosis and fibrosis with heart failure and mortality
Source: Cardiovasc Diabetol. 2021 Sep 28;20:197. doi: 10.1186/s12933-021-01374-8 (PMC8479901; doi:10.1186/s12933-021-01374-8)
Supplement: Supplementary file 1 — Additional file 1: Table S1. Baseline characteristics of study population according to BARD score in patients with NAFLD (defined by FLI ≥ 60) (n = 72,292). Table S2: Hazard ratios and 95% confidence intervals for the incident heart failure, hospitalization for heart failure, all-cause mortality and cardiovascular mortality for FLI ≥ 60 compared with 20 ≤ FLI < 60. Figure S1. Subgroup analysis for incident heart failure in patients with NAFLD (FLI ≥ 60) compared to those without NAFLD (FLI < 20). Figure S2. Subgroup analysis for all-cause death in patients with NAFLD (FLI ≥ 60) compared to those without NAFLD (FLI < 20). [file 12933_2021_1374_MOESM1_ESM.docx]

**Additional file 1**

**The association of hepatic steatosis and fibrosis with heart failure and mortality**

Jiyun Park MD^1,^**^†^**, Gyuri Kim MD, PhD^1,^**^†^**, Hasung Kim, BA^2^, Jungkuk Lee, MS^2^, You-Bin Lee MD^1^, Sang-Man Jin MD PhD^1^, Kyu Yeon Hur MD, PhD^1^, Jae Hyeon Kim MD, PhD^1,3,*^

^1^Division of Endocrinology and Metabolism, Department of Medicine, Samsung Medical Center, Sungkyunkwan University School of Medicine, Seoul, Republic of Korea

^2^Data Science Team, Hanmi Pharm. Co. Ltd., Seoul

^3^Department of Clinical Research Design and Evaluation, Samsung Advanced Institute for

Health Sciences and Technology, Seoul, Republic of Korea

^*^These two authors contributed equally

**^†^Correspondence:**

Jae Hyeon Kim, MD, PhD

Division of Endocrinology and Metabolism, Department of Medicine, Samsung Medical Center, Sungkyunkwan University School of Medicine

81, Irwon-ro, Gangnam-gu, Seoul, 06351, Republic of Korea.

Email: jaehyeon@skku.edu

Phone: +82-2-3410-1580

**Additional Tables**

**Table S1** Baseline characteristics of study population according to BARD score in patients

with NAFLD (defined by FLI ≥ 60) (n = 72,292)

|  | **BARD < 2** | **BARD ≥ 2** | **p-value** |
| --- | --- | --- | --- |
| n (%) | 28411 (39.3) | 43881 (60.7) |  |
| Age (years) | 49.15 ± 8.46 | 54.86 ± 10.37 | < 0.001 |
| Men [n (%)] | 22957 (80.8) | 26602 (60.62) | < 0.001 |
| Income level lowest 25%  [n (%)] | 6120 (21.54) | 10874 (24.78) | < 0.001 |
| Current smoker  [n (%)] | 10301 (36.26) | 11073 (25.23) | < 0.001 |
| Regular exercise  [n (%)] | 4699 (16.54) | 8379 (19.09) | < 0.001 |
| Body weight (kg) | 77.79 ± 9.86 | 75.3 ± 9.98 | < 0.001 |
| BMI (kg/m^2^)^a^ | 27.79 ± 2.79 | 28.31 ± 3.22 |  |
| Waist circumference  (cm)^a^ | 91.83 ± 6.46 | 92.65 ± 6.91 |  |
| In men | 91.91 ± 6.2 | 92.18 ± 6.56 |  |
| In women | 91.48 ± 7.45 | 93.36 ± 7.36 |  |
| SBP (mmHg) | 129.08 ± 14.8 | 131.61 ± 15.96 | < 0.001 |
| DBP (mmHg) | 81.28 ± 10.29 | 81.83 ± 10.53 | < 0.001 |
| Fasting plasma glucose  (mg/dl) | 105.23 ± 29.63 | 111.61 ± 36.38 | < 0.001 |
| AST (IU/L) | 32.51 ± 21.15 | 30.99 ± 26.18 | < 0.001 |
| ALT (IU/L) | 53.26 ± 40.01 | 30.71 ± 22.4 | < 0.001 |
| GGT (IU/L)^a^ | 76.03 ± 67.66 | 64.22 ± 75.5 |  |
| Total cholesterol (mg/dl) | 218.58 ± 45.55 | 214.38 ± 43.93 | < 0.001 |
| Triglyceride (mg/dl)^a^ | 256.63 ± 161.04 | 264.44 ± 205.85 |  |
| HDL-C (mg/dl) | 48.05 ± 30.39 | 52.55 ± 48.37 | < 0.001 |
| LDL-C (mg/dl) | 125.62 ± 104.96 | 121.31 ± 113.43 | < 0.001 |
| eGFR (ml/min/1.73m^2^) | 72.94 ± 21.7 | 72.85 ± 21.93 | 0.601 |
| Comorbidities [n (%)] |  |  |  |
| Hypertension | 11474 (40.39) | 23882 (54.42) | < 0.001 |
| Dyslipidemia | 10318 (36.32) | 17287 (39.4) | < 0.001 |
| Diabetes mellitus^b^ | 3159 (11.12) | 10918 (24.88) |  |
| Metabolic syndrome^c^ | 21195 (74.6) | 35126 (60.7) | < 0.001 |

Continuous variables are expressed as mean ± standard deviation. Categorical data are presented as frequencies and percentages.

^a^p-values are not provided because these variables are included in the equation for FLI.

^b^p-values are not provided because this variable is included in the equation for BARD score.

^c^Metabolic syndrome was defined as a patient presenting with three or more of the following five risk factors: Waist circumference ≥ 90 in men, ≥ 80 in women; triglyceride≥150 mg/dl; HDL < 40 in men, < 50 in women; blood pressure ≥130/≥85; fasting glucose ≥ 100 mg/dl.

AST, alanine aminotransferase; ALT, aspartate aminotransferase; BMI, body mass index;

DBP, diastolic blood pressure; eGFR, estimated glomerular filtration rate; GGT, gamma-glutamyl transferase; HDL, high-density lipoprotein; LDL-C, low-density lipoprotein; SBP, systolic blood pressure.

**Table S2** Hazard ratios and 95% confidence intervals for the incident heart failure, hospitalization for heart failure, all-cause mortality and cardiovascular mortality for FLI ≥ 60 compared with 20 ≤ FLI < 60.

|  | **Model 1** | | | | **Model 2** | | | **Model 3** | | | | | **Model 4** | | | |
| --- | --- | --- | --- | --- | --- | --- | --- | --- | --- | --- | --- | --- | --- | --- | --- | --- |
|  | **Events (n)** | **HR** | **95% CI** | **p value** | **HR** | **95% CI** | **p value** | | **HR** | **95% CI** | **p value** | **HR** | | **95% CI** | **p value** |  |
| iHF |  |  |  |  |  |  |  | |  |  |  |  | |  |  |  |
| 20 ≤ FLI < 60 | 13,146 | Ref. |  |  | Ref. |  |  | | Ref. |  |  | Ref. | |  |  |  |
| FLI ≥ 60 | 4,131 | 1.219 | 1.177-1.262 | <0.001 | 1.287 | 1.238-1.338 | <0.001 | | 1.272 | 1.223-1.323 | <0.001 | 1.132 | | 1.088-1.178 | <0.001 |  |
| hHF |  |  |  |  |  |  |  | |  |  |  |  | |  |  |  |
| 20 ≤ FLI < 60 | 5,630 | Ref. |  |  | Ref. |  |  | | Ref. |  |  | Ref. | |  |  |  |
| FLI ≥ 60 | 1,794 | 1.240 | 1.176-1.308 | <0.001 | 1.462 | 1.378-1.551 | <0.001 | | 1.422 | 1.340-1.509 | <0.001 | 1.269 | | 1.195-1.348 | <0.001 |  |
| All-cause mortality | |  |  |  |  |  |  | |  |  |  |  | |  |  |  |
| 20 ≤ FLI < 60 | 10,752 | Ref. |  |  | Ref. |  |  | | Ref. |  |  | Ref. | |  |  |  |
| FLI ≥ 60 | 3,058 | 1.040 | 0.926-1.168 | 0.505 | 1.403 | 1.237-1.592 | <0.001 | | 1.357 | 1.195-1.541 | <0.001 | 1.19 | | 1.047-1.353 | 0.008 |  |
| CV mortality |  |  |  |  |  |  |  | |  |  |  |  | |  |  |  |
| 20 ≤ FLI < 60 | 1,351 | Ref. |  |  | Ref. |  |  | | Ref. |  |  | Ref. | |  |  |  |
| FLI ≥ 60 | 364 | 1.098 | 1.055-1.143 | <0.001 | 1.486 | 1.422-1.553 | <0.001 | | 1.431 | 1.369-1.495 | <0.001 | 1.331 | | 1.273-1.392 | <0.001 |  |

Model 1: Crude.

Model 2: Age, sex and body weight.

Model 3: Model 2 + alcohol drinking, smoking, regular exercise, and income status.

Model 4: Model 3 + hypertension, diabetes mellitus, dyslipidemia, and estimated glomerular filtration rate.

CI, confidence interval; CV mortality, cardiovascular mortality; HR, hazard ratio; iHF, incident heart failure; hHF, incident hospitalized heart failure

**Additional Figures**





**Fig. S1** Subgroup analysis for incident heart failure in patients with NAFLD (FLI ≥ 60 ) compared to those without NAFLD (FLI < 20).

DM, diametes mellitus; HR, hazard ratio; HTN, hypertension.





**Fig. S2** Subgroup analysis for all-cause death in patients with NAFLD (FLI ≥ 60 ) compared to those without NAFLD (FLI < 20).

DM, diametes mellitus; HR, hazard ratio; HTN, hypertension.
